# Prognostic role of microscopically positive margins for primary gastrointestinal stromal tumors: a systematic review and meta-analysis

**DOI:** 10.1038/srep21541

**Published:** 2016-02-19

**Authors:** Xiaofei Zhi, Baofei Jiang, Junbo Yu, Oluf Dimitri Røe, Jun Qin, Qingfeng Ni, Luning Sun, Meirong Xu, Jianwei Zhu, Lilin Ma

**Affiliations:** 1 Department of General Surgery, the Affiliated Hospital of Nantong University, 20 Xisi Road, Nantong 226001, China; 2 Department of General Surgery, Huai’an First People’s Hospital, Nanjing Medical University, 6 West Beijing Road, Huai’an 223001, China; 3 Emergency Department, the Affiliated Hospital of Nantong University, 20 Xisi Road, Nantong 226001, China; 4 Clinical Cancer Research Center, Aalborg University Hospital, Clinical Institute, Aalborg, Denmark; 5 Cancer Clinic, Levanger Hospital, Nord-Trøndelag Health Trust, Levanger, Norway; 6 Department of Cancer Research and Molecular Medicine, Norwegian University of Science and Technology, Trondheim, Norway; 7 Research Division of Clinical Pharmacology, the First Affiliated Hospital of Nanjing Medical University, 300 Guangzhou Road, Nanjing 210029, China

## Abstract

The impact and management of microscopically positive margins in gastrointestinal stromal tumors (GISTs) remain unclear. The aim of this study is to estimate the prognostic value of surgical margins for disease-free survival (DFS) and overall survival (OS) in patients with primary GISTs. Twelve studies with 1985 GIST patients were included. The overall recurrence rate in R1 resection and R0 resection group was 0.364 (95% CI 0.299–0.429) and 0.296 (95% CI 0.161–0.430), respectively. Meta-analysis confirmed that a microscopically positive margin could significantly impact the disease-free survival (HR 1.596, 95% CI 1.128–2.258; I^2^ = 37.5%, P value = 0.091), but had no influence on overall survival (HR 1.430, 95% CI 0.608–3.363; I^2^ = 60.8%, P value = 0.013). Importantly, subgroup analysis revealed that adjuvant imatinib treatment could attenuate the risk of recurrence for primary GIST patients who received R1 resection. (HR 1.308, 95% CI 0.583–2.935; I^2^ = 53.2%, P value = 0.074). The level of evidence achieved in this study was “moderate” for DFS and “low” for OS. In conclusion, this study revealed that a microscopically positive margin is an unfavorable prognostic factor for GIST patients with R1 resection, and adjuvant imatinib treatment is proved to be effective.

Gastrointestinal stromal tumors (GISTs) are the most common of the mesenchymal tumors, which probably arise from the interstitial cells of Cajal^1^. One of the most prominent characteristics of GISTs is the malignant potential that ranges from a benign behavior to aggressive sarcomas. To evaluate the malignant potential of GISTs, some criteria were established as determined by tumor size, location and number of mitoses, such as NIH criteria^2^, modified NIH criteria^3^ and AFIP criteria^4^. However, increasing evidence show that some other factors, for example sex^5^, genotype^6^,^7^, immune infiltrates^8^, as well as positive surgical margins^9^,^10^, could play an important role in the prognosis of GISTs.

It is a general consensus that surgical excision is the definitive treatment for primary GISTs without peritoneal seeding or metastasis. The goal of surgical treatment is complete gross resection with an intact pseudocapsule and negative microscopic margins (R0), according to the ESMO (European Society for Medical Oncology) and NCCN (National Comprehensive Cancer Network) guidelines^11^,^12^. Notably, apart from three central parameters (tumor size, number of mitosis and tumor location), two additional prognostic factors (surgical margins and tumor rupture) were added in the 2012 edition of ESMO guideline^13^. However, surgical margins were removed in the 2014 edition^11^, on the basis of some emerging evidence. In a prospective randomized series, the majority of patients with an R1 resection did not experience a recurrence in absence of tumor rupture and there was no statistically significant difference in recurrence rate compared with R0 resection^14^. Therefore, given that GISTs often abut some vital structures such as gastroesophageal junction, duodenal papilla as well as retroperitoneal vessels, R1 margins are acceptable especially for low-risk lesions when R0 resection might imply major functional sequelae^11^,^14^. However, other reports support positive microscopic margins (R1) as a main prognostic factor of tumor recurrence^15^,^16^.

These controversial results present challenges to the therapeutic strategies after R1 surgery. The management of R1 resection still remains undefined. The NCCN guideline reported no evidence of a need for re-excision, while the ESMO guideline suggested that re-excision may be an option on the condition that the original site of margins can be found and major functional sequelae are not foreseen. Therefore, it is necessary to perform a systematic and comprehensive meta-analysis to validate the prognostic value of surgical margins and the clinical impact of R1 resection.

In this study, we sought to conduct a meta-analysis to estimate the prognostic value of surgical margins for disease-free survival (DFS) and overall survival (OS) in patients with primary GISTs. In addition, we also comprehensively appraised the quality of evidence and recommended the evidence with Grading of Recommendations Assessment, Development and Evaluation (GRADE) to facilitate clinical decision-making.

## Results

### Study selection and characteristics

**Figure 1:**
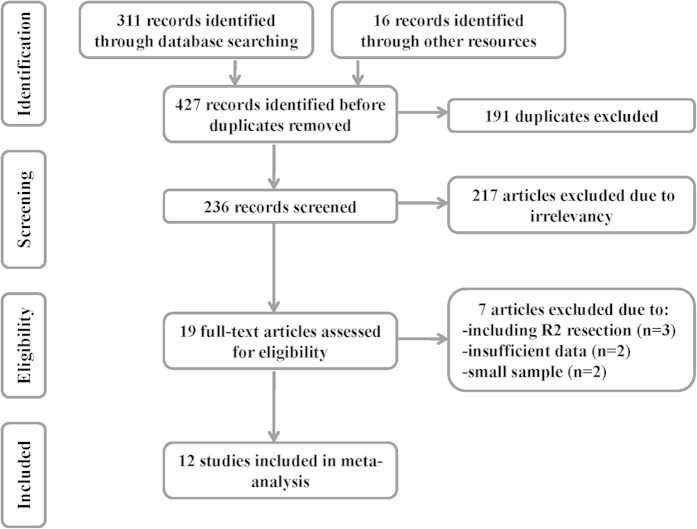
Flow chart of the study selection.

The initial and updated searches together identified 427 records. Upon further review, 19 articles evaluating the prognostic value of surgical margins in patients with primary GISTs were considered eligible. Of the 19 articles, 2 were excluded due to the insufficient reported data for the estimation of HR, 3 were excluded because they put R1 resection and R2 resection together, and 2 were excluded due to the small sample size (there are only 2 cases with R1 margin in both studies)^17^,^18^. Finally, a total of 12 studies^14^,^15^,^16^,^19^,^20^,^21^,^22^,^23^,^24^,^25^,^26^,^27^ were included in this meta-analysis. Literatures screening process was shown in Fig. 1.

The characteristics of included studies are shown in Table 1. In total, 12 studies including 1985 patients were included in the pooled analysis. DFS was obtained in 11 studies, and OS was obtained in 8 studies. All studies had minimum 5 years follow-up period. The rate of R1 resection ranges from 3.5–33.3% (pooled rate 11.9%, 95% CI 8.3–15.4%). Adjuvant imatinib treatment was given in 5 of the studies. The quality of study assessed by Newcastle-Ottawa quality assessment scale ranged from seven to eight, with high value indicating eligible methodology (Table S1).

### Recurrence rate

**Figure 2:**
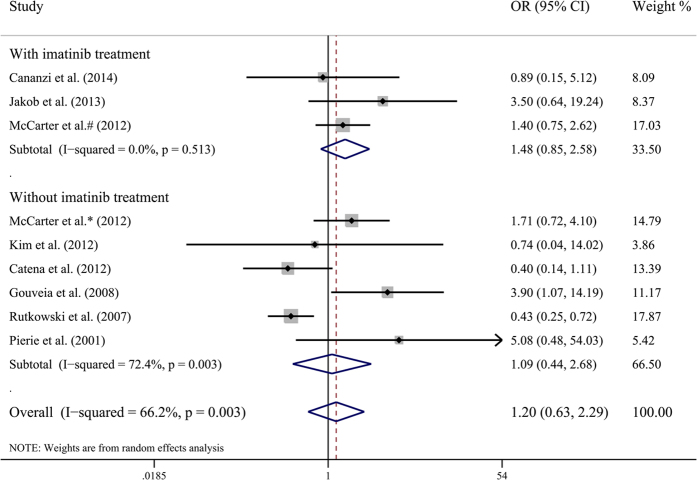
Meta-analysis and stratified analysis of odds ratios of R1 resection for tumor recurrence. Each study is shown by the name of the first author and the odds ratio (OR) with 95% confidence intervals (CIs).

The overall recurrence rate in R1 resection and R0 resection group was 0.364 (95% CI 0.299–0.429) and 0.296 (95% CI 0.161–0.430), respectively. Although the recurrence rate in R1 resection group was slightly higher than that in R0 resection group, meta-analysis revealed that R1 resection did not increase the recurrence rate of GISTs (OR by fixed-effects model 0.891, 95% CI 0.653–1.215; OR by random-effects model 1.203, 95% CI 0.632–2.287; I^2^ = 66.2%, P value = 0.003) (Fig. 2). To explore the source of heterogeneity, meta-regression and subgroup analysis were performed. Adjuvant imatinib treatment was not the source of heterogeneity (Table 2). Sensitivity analysis indicated that pooled ORs were not significantly influenced by omission of any single study.

### Disease-free survival

**Figure 3:**
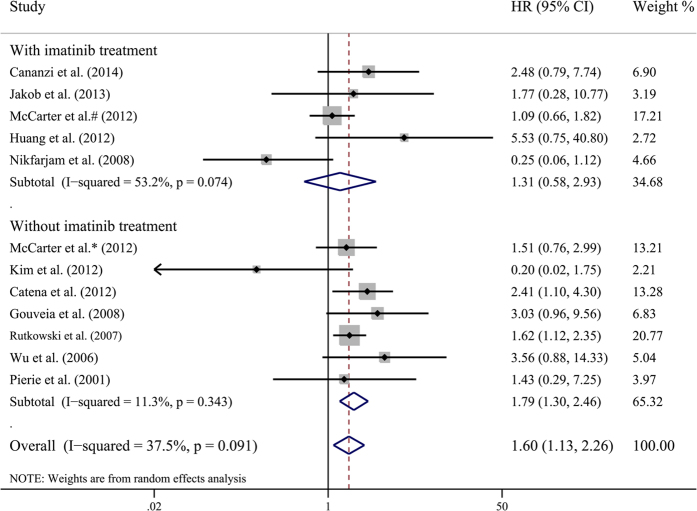
Meta-analysis and stratified analysis of hazard ratios of R1 resection for disease-free survival. Each study is shown by the name of the first author and the hazard ratio (HR) with 95% confidence intervals (CIs).

Meta-analysis using a random-effects model indicated that R1 resection had an unfavorable DFS compared with R0 resection (HR 1.596, 95% CI 1.128–2.258; I^2^ = 37.5%, P value = 0.091), which was consistent with the results of the fixed-effects model (HR 1.567, 95% CI 1.246–1.969) (Fig. 3). To determine whether other factors had an influence on the HR of DFS, we carried out subgroup analysis and meta-regression analysis. Table 3 demonstrates the overall and stratified analysis. Notably, in the adjuvant imatinib treatment subgroup, the patients with R1 resection showed no significant difference of DFS compared with R0 resection (HR 1.308, 95% CI 0.583–2.935; I^2^ = 53.2%, P value = 0.074). In the subgroup without adjuvant imatinib treatment, DFS of R1 resection still remained poor (HR 1.758, 95% CI 1.338–2.310; I^2^ = 11.3%, P value = 0.343). These results indicate that adjuvant imatinib treatment could attenuate the risk of recurrence for primary GIST patients who received R1 resection. Sensitivity analysis indicated that pooled HR was not significantly influenced by omission of any single study.

### Overall survival

**Figure 4:**
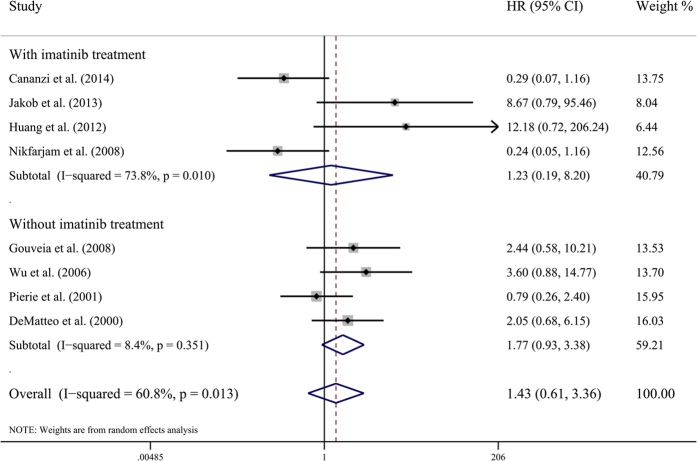
Meta-analysis and stratified analysis of hazard ratios of R1 resection for overall survival. Each study is shown by the name of the first author and the hazard ratio (HR) with 95% confidence intervals (CIs).

Eight studies including 553 GIST patients assessed overall survival (Table 4). Although R1 resection showed a tendency of poor OS, it failed to get a statistically significant HR (HR by fixed-effects model 1.283, 95% CI 0.770–2.138; HR by random-effects model 1.430, 95% CI 0.608–3.363; I^2^ = 60.8%, P value = 0.013) (Fig. 4). When stratified by adjuvant imatinib treatment, there was no significant difference between R1 resection and R0 resection in adjuvant imatinib treatment subgroup (HR 1.232, 95% CI 0.185–8.204; I^2^ = 73.8%, P value = 0.01), or in subgroup without adjuvant treatment (HR 1.755, 95% CI 0.947–3.254; I^2^ = 8.4%, P value = 0.351). Sensitivity analysis indicated that pooled HR was not significantly influenced by omitting any single study.

### Publication bias

No evidence of publication bias was detected by either Begg’s or Egger’s test for OR of recurrence (Begg’s P = 1.000, Egger’s P = 0.197), HR of DFS (Begg’s P = 0.891, Egger’s P = 0.986), or HR of OS (Begg’s P = 0.322, Egger’s P = 0.350). The shape of the funnel plots did not reveal obvious asymmetry (Supplementary Figure S1).

### Quality of evidence

This meta-analysis contained three outcomes: recurrence rate, disease-free survival and overall survival. The GRADE system evidence for each outcome level and reasons for upgrade and downgrade are shown in Table 5.

## Discussion

Due to insights in the molecular targeted therapy and improved surgical techniques, treatment of GISTs has developed rapidly. Unpredictable malignant potential and rare lymph node metastasis provided the theoretical basis for the concept of minimally invasive surgery for GISTs. Consequently, several minimally invasive surgical approaches have been introduced, such as local wedge excision, laparoscopic surgery, endoscopic enucleation and related variations of this technique. However, there is a fine balance between complete resection and minimally invasive resection. Sometimes, an R1 resection might be inescapable owing to the following reasons. First, as a neoplasm arising from the mesenchymal tissue, GIST often abuts significant vessels and other vital structures. Thus, to avoid major functional sequelae, organ-preserving surgery is acceptable with a close or microscopically positive margin. Second, DeMatteo *et al.*^27^ suggested that large GISTs that infiltrated the serosa of the bowel wall, might shed cells from anywhere along their surface into the peritoneum. This theory was also supported by the study of Crosby *et al.*^28^. Third, with increasing upper gastrointestinal examination by endoscopy, the incidence of subclinical GIST has been higher than anticipated^29^. These incidentally encountered GISTs are often treated by endoscopic enucleation. However, Tumor enucleation is considered insufficient, given that it may leave behind a tumor-seeded pseudocapsule with R1 resection^1^,^30^. Even though some innovative endoscopic procedures attempte to support enucleation, such as endoscopic full-thickness resection (EFTR)^31^, laparoscopy endoscopy cooperative surgery (LECS)^32^, laparoscopy-assisted endoscopic full-thickness resection (LAEFR)^33^, and non-exposed wall-inversion surgery (NEWS)^34^, the efficacy and safety of those procedures are still assessed in the clinical trials. Fourth, the surgical safe margins are not well defined for GISTs. Most reports indicated that the 1–2 cm margins including the 5 mm of microscopic distance might be adequate in GISTs^35^,^36^. But the guidelines are not conclusive.

This meta-analysis included 12 studies with 1985 surgically treated primary GIST patients. The overall recurrence rate in R1 resection and R0 resection group was 0.364 (95% CI 0.299–0.429) and 0.296 (95% CI 0.161–0.430), respectively. Although some of these studies found that macroscopic tumor-free margins are essential to a good outcome of GIST surgery, but whether the status of microscopic margins could impact the recurrence remains unclear. The pooled results from this meta-analysis confirmed that a microscopically positive margin could significantly impact the disease-free survival (HR 1.596, 95% CI 1.128–2.258; I^2^ = 37.5%, P value = 0.091), but had no influence on overall survival (HR 1.430, 95% CI 0.608–3.363; I^2^ = 60.8%, P value = 0.013). Importantly, subgroup analysis revealed that there was no significant difference of DFS between R1 resection and R0 resection in the era of adjuvant imatinib treatment (HR 1.308, 95% CI 0.583–2.935; I^2^ = 53.2%, P value = 0.074), however, in the subgroup without adjuvant imatinib treatment, R1 resection still remained unfavorable (HR 1.758, 95% CI 1.338–2.310; I^2^ = 11.3%, P value = 0.343). These results indicated that adjuvant imatinib treatment could reduce the risk of recurrence for primary GIST patients who received R1 resection, clearly indicating a need for adjuvant treatment in this group. Although there is no evidence that re-excision could benefit the patients with R1 resection, re-excision is acceptable only if the original site of lesion can be found, and major functional sequelae are not foreseen^11^. Based on the results of this meta-analysis, adjuvant imatinib treatment is proved to be effective for GIST patients with R1 resection. Therefore, we suggest that adjuvant imatinib treatment combined with re-excision might be a standard approach for patients with R1 resection, and adjuvant imatinib treatment alone could also be sufficient for patients who are not suitable to undergo re-excision. The final decision should be carefully made by the multidisciplinary care team taking into account of possible risks and benefits.

The level of evidence achieved in this meta-analysis was assessed by the GRADE approach (Table 5). The critical outcomes: the quality of DFS was “moderate” and the quality of OS was “low”. The important outcome: the quality of recurrence rate was “low”. The levels of these outcomes were confined due to the limited evidence derived from observational studies. In addition, the quality of recurrence rate and OS was degraded due to the observational studies. Therefore, the quality of evidence for R1 resection as an unfavorable prognostic factor of GISTs is acceptable.

Some limitations of this meta-analysis should be discussed. The analysis of the included studies reporting the status of surgical margins needs to account for some confounding variables. The rate of R1 resection seemed to be associated with both tumor size and recurrent risk^14^,^37^. Given that GISTs with “intermediate” or “high” risk are easier to obtain microscopically positive margins, it is difficult to distinguish whether surgical margin or tumor grade is the major factor of recurrence risk for the patients with R1 resection. It is possible that surgical margin and tumor grade have a collinear relation in the analysis of tumor recurrence. To clarify this assumption, further studies with large sample of patients who received R1 resection are needed and analysis of the relevance between status of surgical margins and tumor grade should also performed in those studies. Unfortunately, there is only one study^14^ reporting the risk factors associated with and R1 resection among the studies included in this meta-analysis. Besides, this study proves that adjuvant imatinib treatment is effective in reducing the recurrence rate by the analysis of all patients with R1 resection. However, whether adjuvant imatinib treatment is essential for patients with R1 resection who are “very low” or “low” grade cannot be determined from this meta-analysis.

In conclusion, this meta-analysis revealed that a microscopically positive margin could significantly impact the disease-free survival, but had no influence on overall survival. In addition, adjuvant imatinib treatment could reduce the risk of recurrence for primary GIST patients who received R1 resection. Our results validated the goal of surgery with a negative microscopic margin and determined the management of R1 resection for GISTs.

## Methods

### Literature search

Two investigators (XZ and BJ) performed a systematic literature search in PubMed, EMBASE, Cochrane Library and Web of Science databases (last updated on July 15, 2015), using combinations of the following terms: “gastrointestinal stromal tumors”, “GIST”, and “margins”. Disagreements were resolved through consensus with a third investigator (LM). Reports in English were eligible for inclusion. The bibliographies cited in selected articles were also examined to identify other relevant studies. Conference abstracts were excluded due to the insufficient data reported. All studies were carefully evaluated to identify duplicate data.

### Inclusion criteria

The following criteria were used for the study selection: (1) Participants (P): All patients were diagnosed as primary GIST using pathology and immunohistochemistry. (2) Interventions (I) and comparisons (C): Comparing the prognosis of R1 resection versus R0 resection. (3) Outcomes (O): Disease-free survival (DFS), Overall survival (OS), and microscopically positive margin rate (MPMR). (4) Study design (S): retrospective or prospective study. (5) Sufficient information allowing for estimation of hazard ratios (HRs) and 95% confidence intervals (CIs).

### Qualitative assessment

Quality assessment was performed in each of the acceptable studies in duplicate by independent reviewers (XZ and BJ) using the Newcastle-Ottawa Quality Assessment Scale for cohort studies. Discrepancies were resolved by a third reviewer (LM).

### Data extraction

Two reviewers (XF and JY) independently extracted the required information from all primary studies. If HRs or their 95% CIs were not directly reported in the included studies, they were estimated according to the available survival data using a method reported by Tierney *et al.*^38^. If the trial results were reported in multiple publications, we extracted the data from the article with the strictest methodology and the most complete data.

### Statistical analysis

We used the PRISMA checklist^39^ as protocol of the meta-analysis and followed the guideline (Table S2). For the pooled analysis of the survival data, HRs and their 95% CIs were used. The point estimate of the HR was considered statistically significant at the p < 0.05 level if the 95% CI did not include the value “1”.

Heterogeneity assumption was checked by the chi-square-based Q-test. A *p*-value >0.10 for the Q-test indicates a lack of heterogeneity among studies. We also quantified the effect of heterogeneity using I^2^ test. I^2^ values of <25% may be considered “low,” values of about 50% may be considered “moderate” and values of >75% may be considered “high”^40^. Both fixed-effects (Mantel–Haenszel method) and random-effects (DerSimonian–Laird method) models were used to estimate the pooled HRs/ORs. Given the inherent between-study heterogeneity, the random-effects model was chosen. To explore the heterogeneity between studies better, meta-regression analysis and subgroup analysis were performed. To explore the dynamic trends as studies accumulated over time, cumulative meta-analysis was performed by date of publication. Sensitivity analyses were conducted to validate whether modification of inclusion criteria affected the results. Potential publication bias was estimated by the funnel plot and Egger’s linear regression test. All analyses were carried out using STATA version 10.0 (Stata Corporation, College Station, TX). Two-sided *p* < 0.05 was considered statistically significant.

### Grading the quality of evidence

The GRADE approach was used to interpret and validate the results^41^. The GRADEprofiler version 3.6 was used to create the evidence profile. For each outcome, we graded the quality of the studies at four levels as “very low”, “low”, “moderate” and “high” on the basis of risk of bias, inconsistency, indirectness, imprecision and publication bias.

## Additional Information

**How to cite this article**: Zhi, X. *et al.* Prognostic role of microscopically positive margins for primary gastrointestinal stromal tumors: a systematic review and meta-analysis. *Sci. Rep.* **6**, 21541; doi: 10.1038/srep21541 (2016).

## Supplementary Material

Supplementary Information

## Figures and Tables

**Table 1:** Main characteristics of the included studies.

Study	Year	Sample size	R0 resection	Recurrence in R0 resection	R1 resection	Recurrence in R1 resection	Adjuvant imatinib treatment	Survival analysis	Outcomes	Follow-up (median, range)	Quality score
Cananzi *et al.*	2014	92	86	31	6	2	Yes	Kaplan–Meier	DFS, OS	41, 2–145 months	7
Jakob *et al.*	2013	36	24	3	12	4	Yes	Kaplan–Meier	DFS, OS	41, 3–110 months	7
McCarter *et al.*	2012	817	745	204	72	26	Yes	Kaplan–Meier	DFS	49 months	8
Kim *et al.*	2012	136	122	5	14	0	No	Kaplan–Meier	DFS	29, 3–106 months	7
Huang *et al.*	2012	85	82	NR	3	NR	Yes	Kaplan–Meier	DFS, OS	41, 3–100 months	7
Catena *et al.*	2012	151	132	71	19	6	No	Cox regression	DFS	101, 11–132 months	7
Nikfarjam *et al.*	2008	40	35	NR	5	NR	Yes	Kaplan–Meier	DFS, OS	24, 1–74 months	7
Gouveia *et al.*	2008	96	78	7	18	5	No	Cox regression	DFS, OS	42, 1–206 months	7
Rutkowski *et al.*	2007	328	253	151	75	29	No	Cox regression	DFS	31, 4–292 months	7
Wu *et al.*	2006	85	81	NR	4	NR	No	Cox regression	DFS, OS	33, 5–202 months	7
Pierie *et al.*	2001	39	35	13	4	3	No	Cox regression	DFS, OS	38, 1–159 months	7
DeMatteo *et al.*	2000	80	65	NR	15	NR	No	Kaplan–Meier	OS	24, 1–175 months	7

DFS, disease-free survival; OS, overall survival; NR, not reported.

**Table 2:** Meta-regression and stratified analysis of pooled odds ratios of R1 resection for tumor recurrence.

Stratified analysis	No. of studies	No. of patients	Pooled OR (95% CI)		Heterogeneity		Meta-regression	
			Fixed	Random	I^2^	*p*-value	Residual I^2^	*p*-value
Overall	9	1695	0.891 (0.653–1.215)	1.203 (0.632–2.287)	66.2%	0.003^*^		
Adjuvant imatinib treatment							63.98%	0.592
Yes	3	592	1.474 (0.849–2.558)	1.477 (0.846–2.579)	0.0%	0.513		
No	6	1103	0.715 (0.490–1.041)	1.092 (0.445–2.683)	72.4%	0.003^*^		

OR, odds ratios; CI, confidence interval; Fixed, fixed-effects model; Random, random-effects model; ^*^indicating heterogeneity.

**Table 3:** Meta-regression and stratified analysis of pooled hazard ratios of R1 resection for disease-free survival.

Stratified analysis	No. of studies	No. of patients	Pooled HR (95% CI)		Heterogeneity		Meta-regression	
			Fixed	Random	I^2^	*p*-value	Residual I^2^	*p*-value
Overall	12	1905	1.567 (1.246–1.969)	1.596 (1.128–2.258)	37.5%	0.091^*^		
Adjuvant imatinib treatment							34.47%	0.252
Yes	5	717	1.193 (0.784–1.815)	1.308 (0.583–2.935)	53.2%	0.074^*^		
No	7	1188	1.758 (1.338–2.310)	1.785 (1.298–2.456)	11.3%	0.343		
Sample size							40.36%	0.523
>100	5	1432	1.495 (1.161–1.924)	1.472 (1.024–2.116)	39.6%	0.157		
<100	7	473	1.940 (1.132–3.325)	1.897 (0.922–3.902)	41.5%	0.115		

HR, hazard ratios; CI, confidence interval; Fixed, fixed-effects model; Random, random-effects model; ^*^indicating heterogeneity.

**Table 4:** Meta-regression and stratified analysis of pooled hazard ratios of R1 resection for overall survival.

Stratified analysis	No. of studies	No. of patients	Pooled HR (95% CI)		Heterogeneity		Meta-regression	
			Fixed	Random	I^2^	*p*-value	Residual I^2^	*p*-value
Overall	8	553	1.283 (0.770–2.138)	1.430 (0.608–3.363)	60.8%	0.013^*^		
Adjuvant imatinib treatment							58.96%	0.667
Yes	4	253	0.651 (0.262–1.616)	1.232 (0.185–8.204)	73.8%	0.010^*^		
No	4	300	1.755 (0.947–3.254)	1.769 (0.926–3.380)	8.4%	0.351		

HR, hazard ratios; CI, confidence interval; Fixed, fixed-effects model; Random, random-effects model; ^*^indicating heterogeneity.

**Table 5:** GRADE evidence profile.

Quality assessment							No of patients		Effect		Quality	Importance
No of studies	Design	Risk of bias	Inconsistency	Indirectness	Imprecision	Other considerations	R1 resection	R0 resection	Relative (95% CI)	Absolute		
Recurrence rate (follow-up 8.8–24 years)												
9	observational studies	no serious risk of bias	serious^1^	no serious indirectness	no serious imprecision	increased effect for RR ~1^2^	75/220 (34.1%)	485/1475 (32.9%)	OR 1.203 (0.632 to 2.287)	42 more per 1000 (from 92 fewer to 200 more)	⊕⊕ΟΟ Low	Critical
Disease-free survival (follow-up 6.1–24 years)												
12	observational studies	no serious risk of bias	no serious inconsistency	no serious indirectness	no serious imprecision	increased effect for RR ~1^2^	232	1673	HR 1.596 (1.128 to 2.258)	—	⊕⊕Ο Moderate	Critical
Overall survival (follow-up 6.1–17 years)												
8	observational studies	no serious risk of bias	serious^1^	no serious indirectness	no serious imprecision	increased effect for RR ~1^2^	67	486	HR 1.430 (0.068 to 3.363)	—	⊕⊕ΟΟ Low	Important

^1^The statistical test for heterogeneity showed a low P value.

^2^Numerous specimen processing variables when assessing positive margins might influence the rate of positive margins.
